# Factorial Validity and Measurement Invariance of the Slovene Version of the Cultural Intelligence Scale

**DOI:** 10.3389/fpsyg.2018.01499

**Published:** 2018-08-21

**Authors:** Eva Boštjančič, Luka Komidar, Richard B. Johnson

**Affiliations:** ^1^Department of Psychology, Faculty of Arts, University of Ljubljana, Ljubljana, Slovenia; ^2^Department of Leadership Studies, Indiana Wesleyan University, Marion, OH, United States

**Keywords:** cultural intelligence scale, cultural intelligence, factorial validity, measurement invariance, general cultural intelligence factor

## Abstract

This study examined the factorial validity of the Slovene version of the cultural intelligence scale (CQS) in a representative sample of 1,000 Slovenian participants (49% were female). The results of confirmatory factor analysis supported the factorial validity of the Slovene CQS and the existence of a general (second-order) cultural intelligence factor. The four scales and the overall (general) CQS scale showed satisfactory internal consistency. The results of multiple-group confirmatory factor analyses supported the hypotheses of partial measurement invariance across gender, and full measurement invariance across type of settlement (urban vs. rural).

## Introduction

Cultural intelligence (CQ) refers to the capability to adapt effectively to new cultural contexts (Earley and Ang, 2003). It relates to effectiveness in situations of cultural plurality (Ang and Van Dyne, 2008) in which individuals with high cultural intelligence are culturally competent and have a broad repertoire of (meta)cognitive, behavioral and motivational abilities that enable them to work effectively with members of different cultures and adapt to foreign environments.

The cultural intelligence scale (CQS) (Ang et al., 2007) is the most widely used self-report instrument for measuring the four dimensions of cultural intelligence (i.e., metacognitive, cognitive, motivational, and behavioral; the CQS items in English are provided in **Supplementary Table 1**). Van Dyne et al. (2008) showed that the structure of the CQS is stable over time, across multiple samples, and various countries. Although Ang et al. discussed an overall CQ as an aggregate multidimensional construct, none of the later validation studies that reported moderately high to high correlations between the scales (e.g., Ward et al., 2009; Imai and Gelfand, 2010) examined a second-order model with a general CQ factor. However, this second-order model was tested in a study on cross-border leadership effectiveness by Rockstuhl et al. (2011), but they only reported the (adequate) fit statistics of the second-order model and did not provide any information about the relationships between this (general) second-order and the four first-order factors. Regarding the criterion (concurrent and predictive) validity of the CQS, numerous and diverse studies have consistently showed that the CQS scale-scores represent important individual- and intercultural team-level predictors of various psychological, behavioral, and performance outcomes (for an overview see Leung et al., 2014; Ott and Michailova, 2018).

**Table 1:** Structure of the CQS (average standardized loadings per factor), Cronbach’s alpha coefficients, and correlations between the CQS factors according to the confirmatory factor analysis (*N* = 1,000).

CQS factor	Metacognitive	Cognitive	Motivational	Behavioral
*M*(λ)	0.75	0.71	0.72	0.77
Metacognitive	(0.83)			
Cognitive	0.55	(0.86)		
Motivational	0.68	0.66	(0.84)	
Behavioral	0.60	0.59	0.76	(0.88)


*λ = standardized loading (all standardized loadings are presented in **Supplementary Table 1**). Cronbach’s α coefficients are in parenthesis in the diagonal. All correlations were statistically significant, p < 0.01.*

The CQS has already been used in Slovenia in the context of the links between knowledge and cultural intelligence (Gotnik Urnault, 2014), the role of cultural intelligence for creativity in a culturally diverse environment (Bogilović and Škerlavaj, 2016), and associations between cultural intelligence of Slovene students and their international experiences (Jakovljević et al., 2016). Because previous attempts at Slovene adaptation of the CQS included only (improper) translation and did not examine the psychometric properties of the CQS, the goals of this study were to conduct a more thorough translation, and to assess the factorial validity of the CQS in a representative Slovene sample. We also examined measurement invariance of the CQS across gender and type of settlement (urban vs. rural).

## Materials and Methods

### Participants

The sample included 1,000 participants from Slovenia; 49% were female (*M*_age_ = 52.2 years, *SD*_age_ = 14.7), and 51% were male (*M*_age_ = 44.7 years, *SD*_age_ = 15.9). Their mean age was 48.7 years (*SD* = 15.8). Approximately half of the participants (50.7%) had an elementary or vocational qualification, 35.4% had a secondary or higher vocational qualification, and 13.8% had a bachelor’s degree or higher. One third (33.3%) of participants came from urban settlements in Slovenia (i.e., densely populated settlements with at least 2,000 inhabitants). Mean age of urban and rural participants was 49.6 years (*SD* = 15.7) and 48.2 years (*SD* = 15.7), respectively.

### Instrument

The cultural intelligence scale (Ang et al., 2007) is a 20-item self-report scale measuring metacognitive (α = 0.76; the reported alphas are from Ang et al.), cognitive (α = 0.80), motivational (α = 0.79), and behavioral (α = 0.82) aspects of cultural intelligence on a 7-point Likert scale (1 = strongly disagree, 7 = strongly agree). The items of the CQS in English are provided in **Supplementary Table 1**.

### Procedure

We used the established systematic approach of a familiarity- and recognisability-driven adaptation of a questionnaire (Malda et al., 2008) when translating the CQS to Slovene. The translation process was done according to expert recommendations (e.g., Hambleton and De Jong, 2003) and thus included several rounds of translation and back translation using two experts in organizational psychology and two in sociology, as well as a bilingual language expert. The translated items were then subjected to focus groups discussions pertaining to the comprehensibility of items and specific characteristics of Slovene citizens’ perceptions regarding cross-cultural interaction. Based on the opinions of 19 participants, we reworded some items to make them more suitable for the Slovene cultural and linguistic environment.

Data was collected by a marketing research agency Ipsos. They used computer-assisted telephone interviewing and employed stratified sampling technique to ensure representativeness of the sample for the Slovene general population.

This study was carried out in accordance with the recommendations of ICC/ESOMAR International Code on Market, Opinion and Social Research and Data Analytics (ESOMAR, 2016). According to these ICC/ESOMAR guidelines, this type of study represents market research and does not require the approval of an ethics committee, as this survey was conducted on participants who are considered healthy and not in the medical system. At the beginning of the telephone calls, the participants were provided with a detailed description of the study. All participants gave spoken informed consent in accordance with the Declaration of Helsinki.

### Statistical Analyses

All confirmatory factor analyses (CFAs) were done in M*plus* 7.2 (Muthén and Muthén, 1998–2012). We used the robust MLM estimator, because the majority of items had moderately non-normal distributions; the skewness of the items ranged from –0.74 to 0.29, and the kurtosis values ranged from –0.65 to 0.10. For assessing the goodness of fit of the tested models to the data, we used the CFI, TLI, RMSEA, and SRMR fit indexes. The following cut-off values were considered as indicating adequate fit (Hu and Bentler, 1999): CFI and TLI > 0.95, RMSEA < 0.05, and SRMR < 0.08. We carried out a multiple-group CFA to test the measurement invariance of the CQS. We tested the hypotheses regarding equality of factor loadings (i.e., metric invariance) and equality of indicator intercepts (i.e., scalar invariance). Because using large samples can lead to excessively restrictive test of measurement invariance (via the likelihood ratio test), we used a *p*-value of 0.01 as a criterion for statistical significance, and additionally observed the differences in the CFI between the nested models (ΔCFI larger than 0.002 as indicating lack of measurement invariance; Meade et al., 2008).

## Results

### Factorial Validity of the Slovene CQS

The fit of the 4-factor model to the data was adequate: χ^2^_(164)_ = 409.4, *p* < 0.01, CFI = 0.97, TLI = 0.96, RMSEA = 0.039, SRMR = 0.035. The standardized loadings across all factors ranged from 0.61 to 0.83. **Table 1** shows the average standardized loadings, Cronbach’s α coefficients for each of the four factors, and correlations between the CQS factors according to the CFA. Detailed information about the standardized loadings, items’ means and standard deviations can be found in **Supplementary Table 1**. In sum, the fit indexes and relatively high standardized loadings indicate satisfactory construct (factorial) validity of the Slovene version of the CQS. The CQS scales also showed satisfactory internal consistency.

As can be seen from **Table 1**, the correlations between the four factors were positive and quite high, which is in accordance with the notion of an overall/general factor of CQ (Ang et al., 2007; Thomas et al., 2008; Rockstuhl et al., 2011). Therefore, we also tested the fit of the second-order factor model by specifying the four first-order factors as indicators of a (general) second-order cultural intelligence factor. The fit of the second-order model was adequate: *χ*^2^_(166)_ = 411.2, *p* < 0.01, CFI = 0.97, TLI = 0.96, RMSEA = 0.038, SRMR = 0.035. The standardized weights (*M*[*λ*] = 0.80; **Supplementary Table 1**) for the general CQS factor were high, and the reliability of the total CQS score was excellent (α = 0.93).

### Measurement Invariance of the Slovene CQS Across Gender and Type of Settlement

To test the measurement invariance of the CQS across gender and type of settlement we tested the hypotheses regarding the equality of factor loadings and equality of item intercepts. Regarding gender, the configural models were adequate (**Table 2**). Imposing the constraints of the metric model (equal loadings) did not statistically significantly change the fit of the 4-factor model, while imposing the constraints of the scalar model (equal item intercepts) resulted in a statistically significant change in overall model fit. To achieve partial scalar invariance, we reviewed the modification indexes pertaining to the item intercepts. We consecutively freed the item intercepts associated with the largest modification indexes (MI) until the change in overall model fit was not statistically significant any more.

**Table 2:** Measurement invariance statistics for gender and type of settlement groups, and factor means (structural) invariance model.

							Model difference tests			
Type of invariance	χ^2^	*df*	CFI	TLI	RMSEA	SRMR	Δχ^2^	Δ *df*	*p*	Δ CFI
**Across gender**
Configural	583.0	328	0.964	0.959	0.039	0.040				
Metric (full)	604.6	344	0.963	0.960	0.039	0.043	18.7	16	0.284	–0.001
Scalar (full)	650.3	360	0.959	0.960	0.040	0.044	52.9	16	<0.001	–0.004
Scalar (partial)^a^	627.6	357	0.962	0.960	0.039	0.043	23.0	13	0.042	–0.001
Factor means	641.8	361	0.961	0.959	0.039	0.046	10.88	4	0.028	
**Across type of settlement (urban vs. rural)**
Configural	630.0	328	0.958	0.951	0.043	0.041				
Metric (full)	643.3	344	0.958	0.954	0.042	0.042	5.9	16	0.989	–0.000
Scalar (full)	660.1	360	0.958	0.956	0.041	0.042	11.1	16	0.804	–0.000
Factor means	663.2	364	0.958	0.956	0.041	0.042	0.9	4	0.924	


*Δχ^*2*^ = nested χ^*2*^ difference (Satorra–Bentler scaled chi-square; also referred to as likelihood ratio test [–2Δ LL]); Δ df = degrees of freedom for the model difference test. ^*a*^Free intercepts of the items 8, 5, 17 in the female group.*

We first freed the intercept of the Item 8 (MI = 9.9; see **Supplementary Table 1** for item contents), but the modified scalar model still had a significantly worse fit than the full metric invariance model, Δχ^2^= 38.8, *p* = 0.001 (however, ΔCFI was –0.002 after freeing the intercept of Item 8). The second largest MI was associated with Item 5 (MI = 7.3); freeing this intercept still resulted in a significant change of the model fit, Δχ^2^= 31.5, *p* = 0.005. After freeing the intercept of Item 17 (MI = 6.0), the change in model fit was no longer statistically significant, Δχ^2^= 23.0, *p* = 0.04. To achieve partial scalar invariance, we had to free two item intercepts (out of six) in the cognitive domain [Item 8: (unstandardized) intercept was higher for females (3.56) than males (3.32); Item 5: the intercept was lower for females (3.30) than males (3.52)], and one item intercept (out of five) in the behavioral domain [Item 17: the intercept was higher for females (4.74) than males (4.52)]. In the last step, we also addressed the question whether female and male participants differ in the average level of the CQS factors. This was done by imposing an additional constraint of equal factor means across both gender groups. The change in overall model fit was statistically significant (**Table 2**), which indicated that females and males differ on at least one CQS factor. The tests of factor mean differences revealed that the females exhibited a slightly higher level of the metacognitive aspect of cultural intelligence (Δ*M* = 0.23, *p* < 0.01).

Concerning measurement invariance across type of settlement, imposing the constraints of the metric and scalar models did not result in statistically significant changes in the fit of the 4-factor model (**Table 2**). Imposing the constraints of the factor means invariance model did not lead to a significant change in overall model fit, which means that the participants form urban and rural type of settlements do not differ on any of the CQS factors.

## Discussion

The results of confirmatory factor analysis supported the factorial validity of the Slovene version of the CQS, and the reliabilities of the four scales were satisfactory. Our results and the results of previous validation studies (e.g., Ang et al., 2007; Van Dyne et al., 2008; Imai and Gelfand, 2010) that also found an adequate fit of the 4-factor model to the data indicate that the factorial structure of the CQS is quite robust. Since the correlations between the factors were moderately high, we tested the fit of the second-order model by adding one higher-order (general) CQ factor; the fit of this model was again adequate. The standardized loadings of the four first-order factors indicate that the metacognitive and cognitive factors of CQ are, in relative terms, the least important indicators of the general CQ, while the motivational factor is the most important indicator of the general CQ factor. We suggest that future research concerning the structure of the CQS (and CQ nomological network in general) should also always test this second-order model and further examine the added value of using the general CQ factor for predicting relevant outcome variables.

By using multiple-group CFAs we found partial measurement invariance across gender and full measurement invariance across type of settlement. To achieve partial scalar invariance across gender, we had to free three item intercepts. Partial scalar invariance means that for the majority of CQS items the observed differences in their means between gender groups were due to factor mean differences only. The most affected was the Cognitive scale (two non-invariant item intercepts out of six), so we suggest caution when comparing the means of women and men on Cognitive CQS scale.

Overall, the results of the present study confirmed the robustness of the four-factor CQS structure and provided additional evidence for a general CQ factor. Further validation studies of the Slovene CQS should especially focus on the predictive validity of the CQS against most relevant criterion variables on individual- and intercultural dyad- or team-level.

## Author Contributions

EB and RJ conceived the idea for the study. EB carried out all of the tasks for the translation of the CQS to Slovene [coordinating the (back) translations, conducting focus groups] and gathering the data. LK conducted all of the statistical analyses and wrote the manuscript with the support of EB and RJ.

## Conflict of Interest Statement

The authors declare that the research was conducted in the absence of any commercial or financial relationships that could be construed as a potential conflict of interest.
