# Supplementary material for: Factorial Validity and Measurement Invariance of the Slovene Version of the Cultural Intelligence Scale
Source: Front Psychol. 2018 Aug 21;9:1499. doi: 10.3389/fpsyg.2018.01499 (PMC6110893; doi:10.3389/fpsyg.2018.01499)
Supplement: Supplementary file 1 [file Table_1.docx]

Supplementary Material

Factorial Validity and Measurement Invariance of the Slovene Version of the Cultural Intelligence Scale

Eva Boštjančič, Luka Komidar*, Richard Boyd Johnson

*** Correspondence:** Luka Komidar: [luka.komidar@ff.uni-lj.si](mailto:luka.komidar@ff.uni-lj.si)

# Supplementary Table

**Supplementary Table 1.** Structure of the Cultural Intelligence Scale – CQS (standardized loadings) obtained by the confirmatory factor analyses, and item and scale descriptive statistics (*N* = 1000)

| CQS factor | Standardized  loadings | *M* | *SD* |
| --- | --- | --- | --- |
| Metacognitive |  |  |  |
| 1. I am conscious of the cultural knowledge I use when interacting with people with different cultural backgrounds. | .69 | 5.22 | 1.37 |
| 2. I adjust my cultural knowledge as I interact with people from a culture that is unfamiliar to me. | .77 | 5.27 | 1.47 |
| 3. I am conscious of the cultural knowledge I apply to cross-cultural interactions. | .81 | 5.23 | 1.40 |
| 4. I check the accuracy of my cultural knowledge as I interact with people from different cultures. | .71 | 4.88 | 1.58 |
| Cognitive |  |  |  |
| 5. I know the legal and economic systems of other cultures. | .61 | 3.40 | 1.57 |
| 6. I know the rules (e.g., vocabulary, grammar) of other languages. | .61 | 3.67 | 1.63 |
| 7. I know the cultural values and religious beliefs of other cultures. | .78 | 4.31 | 1.55 |
| 8. I know the marriage systems of other cultures. | .75 | 3.44 | 1.56 |
| 9. I know the arts and crafts of other cultures. | .79 | 3.77 | 1.48 |
| 10. I know the rules for expressing non-verbal behaviors in other cultures. | .74 | 3.71 | 1.52 |
| Motivational |  |  |  |
| 11. I enjoy interacting with people from different cultures. | .73 | 5.05 | 1.68 |
| 12. I am confident that I can socialize with locals in a culture that is unfamiliar to me. | .64 | 4.90 | 1.55 |
| 13. I am sure I can deal with the stresses of adjusting to a culture that is new to me. | .75 | 4.84 | 1.62 |
| 14. I enjoy living in cultures that are unfamiliar to me. | .79 | 5.08 | 1.72 |
| 15. I am confident that I can get accustomed to the shopping conditions in a different culture. | .69 | 4.57 | 1.65 |
| Behavioral |  |  |  |
| 16. I change my verbal behavior (e.g., accent, tone) when a cross-cultural interaction requires it. | .74 | 4.36 | 1.68 |
| 17. I use pause and silence differently to suit different cross-cultural situations. | .73 | 4.67 | 1.56 |
| 18. I vary the rate of my speaking when a cross-cultural situation requires it. | .83 | 4.49 | 1.71 |
| 19. I change my non-verbal behavior when a cross-cultural situation requires it. | .81 | 4.46 | 1.67 |
| 20. I alter my facial expressions when a cross-cultural interaction requires it. | .76 | 4.56 | 1.65 |
|  |  |  |  |
| General CQ factor |  | 4.49 | 1.03 |
| Metacognitive | .74 | 5.15 | 1.18 |
| Cognitive | .72 | 3.72 | 1.19 |
| Motivational | .92 | 4.89 | 1.29 |
| Behavioral | .82 | 4.51 | 1.36 |
